# Multi-omics analysis reveals the mechanism of verbenalin in treating gout via modulating purine metabolism, gut microbiota, and inflammatory pathways

**DOI:** 10.3389/fimmu.2026.1761558

**Published:** 2026-02-20

**Authors:** Yan Xiao, Ting Zhang, Qianglong Chen, Yiqian Zhang, Bingyan Chen, Meiling Wang, Yingjie Zhang, Mingqing Huang, Youxin Su, Jiemei Guo

**Affiliations:** 1 School of Orthopedics and Traumatology, Fujian University of Traditional Chinese Medicine, Fuzhou, Fujian, China; 2 Key Laboratory of Orthopedics & Traumatology of Traditional Chinese Medicine and Rehabilitation, Ministry of Education, Fuzhou, Fujian, China; 3 The Affiliated People's Hospital, College of Pharmacy, Fujian University of Traditional Chinese Medicine, Fuzhou, Fujian, China

**Keywords:** gout, MAPK signaling pathway, multiomics, PI3K-Akt signaling pathway, verbenalin

## Abstract

**Background:**

Gout is a prevalent metabolic disorder characterized by hyperuricemia and inflammation. Verbenalin, an iridoid glycoside from Verbena officinalis, possesses anti-inflammatory properties; however, its therapeutic potential and underlying mechanisms in gout remain underexplored.

**Objective:**

This study aimed to evaluate the pharmacological effects and elucidate the molecular mechanisms of verbenalin in a rat model of gout.

**Methods:**

Hyperuricemia and acute gouty arthritis were induced in rats using potassium oxonate/hypoxanthine and monosodium urate, respectively. Verbenalin was administered orally for 7 days. Therapeutic efficacy was assessed via physical symptom scores (inflammation, gait, swelling), renal/hepatic function indices, and histopathology. Furthermore, a multi-omics strategy integrating transcriptomics, metagenomics, and metabolomics, combined with Western blotting, was employed to investigate the pharmacological mechanisms.

**Results:**

Verbenalin treatment significantly alleviated joint inflammation and swelling while improving gait scores. It effectively lowered serum uric acid (UA), creatinine, and BUN levels, inhibited hepatic xanthine oxidase (XOD) activity, and promoted urinary UA excretion. Histopathological damage in the joints, kidneys, and liver was markedly mitigated. Mechanistically, verbenalin downregulated the expression of urate transporters (URAT1, GLUT9) and inflammatory mediators (NLRP3, IL-1β) by inhibiting the PI3K-AKT and MAPK signaling pathways. Multi-omics analysis further revealed that verbenalin restored gut microbiota diversity and modulated purine metabolism, correlating with reduced UA levels.

**Conclusion:**

These findings demonstrate that verbenalin may exert anti-gout effects through the potential synergy of modulating purine metabolism, shifting gut microbiota composition, and suppressing PI3K-AKT and MAPK inflammatory signaling pathways. This study provides a preliminary scientific basis for further investigation into verbenalin as a prospective multi-target therapeutic candidate.

## Graphical Abstract

**Figure:**
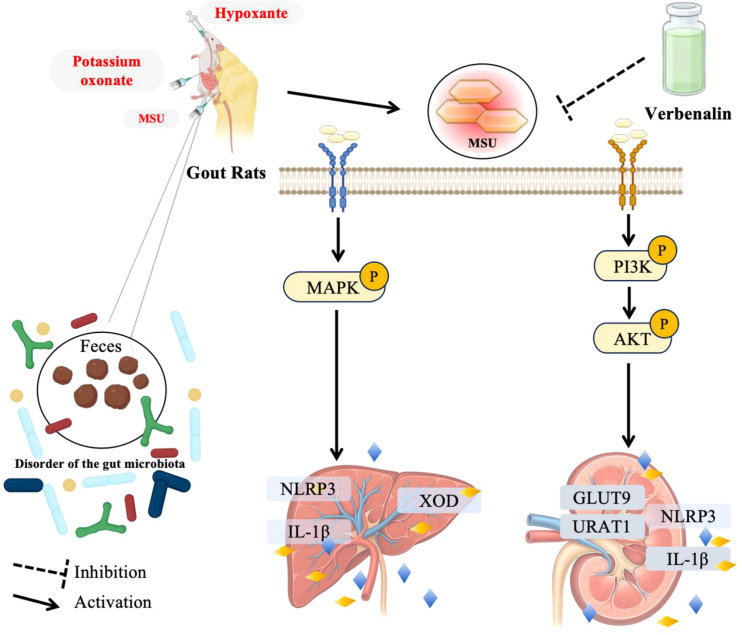
Verbenalin exerts anti-gout effects by synergistically modulating purine metabolism, restoring gut microbiota balance, and suppressing inflammatory signaling (PI3K-AKT, MAPK).

## 1 Introduction

Gout has become an escalating global health concern, particularly in rapidly developing Asia regions (1). Worldwide epidemiological data reveal over 41 million cases, with an alarming annual incidence increase of 5.5% (2). This metabolic disorder, characterized by hyperuricemia (HUA) and recurrent inflammatory arthritis, demonstrates particularly concerning trends in China where prevalence has risen from 0.86% to 2.20% in recent decades, with a notable shift toward younger populations (3, 4). The pathogenesis of this disorder involves two distinct yet interconnected mechanisms: (1) systemic urate homeostasis disruption, resulting from either excessive production or reduced excretion of uric acid (UA), which promotes monosodium urate (MSU) crystal deposition; and (2) NOD-like receptor family pyrin domain containing 3 (NLRP3) inflammasome activation and subsequent pro-inflammatory cascade initiation upon MSU crystal recognition.

Gout management includes rapid treatment of acute flares and effective long-term management. The central strategy for long-term management is reduction of serum urate to a concentration that achieves dissolution of monosodium urate crystals (2). Guide-recommended drugs, including xanthine oxidase (XOD) inhibitors like allopurinol and febuxostat, uricosuric agents such as benzbromarone, which primarily focus on modulating urate metabolism, and anti-inflammatory drugs such as colchicine, etc. However, their clinical utility is constrained by significant limitations (5). These include dose-dependent adverse effects (e.g., hepatotoxicity, hypersensitivity syndrome, gastrointestinal adverse reactions) and single therapeutic effects of the drug. These limitations emphasize the urgent need for safer treatment strategies that concurrently address both HUA and inflammation (6).

Verbenalin, a bioactive iridoid glycoside derived from medicinal plants of the Verbenaceae family, has emerged as a promising multi-target therapeutic agent due to its broad pharmacological properties (7). The compound demonstrates potent anti-inflammatory activity through dual modulation of key inflammatory pathways: (1) inhibition of nuclear factor kappa B (NF-κB) nuclear translocation and subsequent transcriptional activity, and (2) suppression of NLRP3 inflammasome assembly and activation. These mechanisms collectively lead to significant down regulation of pro-inflammatory mediators [tumor necrosis factor-alpha (TNF-α), interleukin-6 (IL-6), interleukin-1 beta (IL-1β)] and reduction in neutrophil recruitment (8, 9). Additionally, verbenalin exhibits remarkable anti-apoptotic effects across multiple pathological contexts. In neurodegenerative disorders, it inhibits mitochondrial cytochrome C release and caspase-3 activation while modulating the Bcl-2/Bax protein balance. Notably, in acute lung injury, verbenalin exerts its protective effects through GPR18 receptor-mediated suppression of macrophage pyroptosis and inhibition of neutrophil extracellular trap (NET) formation, thereby accelerating inflammation resolution (10). Our preliminary investigations have demonstrated verbenalin’s efficacy in MSU-induced macrophage inflammation models, suggesting its potential therapeutic value for gout management (8). However, the precise molecular mechanisms underlying its anti-gout effects and comprehensive *in vivo* therapeutic efficacy remain to be fully elucidated.

Recent advances in bioinformatics and high-throughput sequencing technologies have propelled multi-omics analysis to the forefront of biomedical research, providing unprecedented capabilities for therapeutic target identification and mechanistic investigation of complex diseases (11). These technological breakthroughs have particularly revolutionized the study of traditional Chinese medicine (TCM) compounds, with cutting-edge histomics approaches now enabling systematic exploration of their pharmacological mechanisms. The integration of multi-omics platforms facilitates comprehensive monitoring of TCM-induced modulations across multiple biological levels, including dynamic changes in intracellular metabolite profiles, alterations in metabolic pathway activity, shifts in gut microbiota composition, and genome-wide gene expression regulation. This integrated approach enables systematic mapping of TCM’s complex regulatory networks in disease treatment, provides mechanistic interpretation of its characteristic multi-target, multi-pathway therapeutic actions, and effectively bridges traditional medicine paradigms with modern systems biology frameworks (12–14).

Despite growing interest in verbenalin’s therapeutic potential, current understanding of its pharmacological mechanisms remains fragmentary, particularly regarding its integrated multi-target effects spanning purine metabolic regulation, gut microbiota modulation, and inflammatory signaling pathway inhibition. Therefore, this study aimed to elucidate verbenalin’s therapeutic mechanisms in gout through multi-omics analysis. By integrating transcriptomic, Metagenomicand metabolomics data from gout model rats, we identified potential targets and key signaling pathways of verbenalin while experimentally confirming its anti-inflammatory and urate-lowering effects. These findings could support verbenalin’s therapeutic application for gout and justify its further development as a potential treatment.

## 2 Materials and methods

### 2.1 Experimental materials and reagents

Verbenalin (B21831) was purchased from Shanghai Yuanye Bio-Technology Co., Ltd. (Shanghai, China). Potassium oxonate (PO, P831461), Hypoxanthine (HX, H811076), and MSU (U886060) were purchased from Shanghai Macklin Biochemical Co., Ltd (Shanghai, China). UA (C012-2-1), blood urea nitrogen (BUN, C013-2-1), creatinine (CRE, C011-2-1), XOD (A002-1-1), alanine aminotransferase (ALT, C009-1-1), and aspartate aminotransferase (AST, C010-1-1) assay kits were obtained from Nanjing Jiancheng Bioengineering Institute (Nanjing, China). We obtained the following antibodies: anti-XOD (55156-1-AP), anti-glucose transporter 9 (GLUT9) (26486-1-AP), anti-urate transporter 1 (URAT1) (14937-1-AP), anti-IL-1β (16806-1-AP), anti-phosphoinositide 3-kinase (PI3K) (20584-1-AP), anti-protein kinase B (AKT) (10176-2-AP), and anti-p-AKT (66444-1-Ig) from Proteintech (Wuhan, China); anti-p-MAPK (HA722150) from Hangzhou Hua’an Biotechnology (Hangzhou, China); anti-MAPK (8690S) and anti-NLRP3 (15101S) from Cell Signaling Technology (Massachusetts, USA); anti-p-PI3K (11508) from Signalway Antibody (Maryland, USA); and anti-β-actin (P31029) from TransGen Biotech Co., Ltd. (Beijing, China).

### 2.2 Animal modeling and treatment

All animal experiments received ethical approval (Approval No. FJTCM IACUC, 2024113) from Fujian University of Traditional Chinese Medicine’s Animal Ethics Committee. Procedures strictly complied with the NIH Guide for the Care and Use of Laboratory Animals and rigorously adhered to ARRIVE guidelines.

Sixty male Sprague-Dawley rats (6 weeks old, 220 ± 20 g body weight) were obtained from Shanghai Slyke Laboratory Animal Co., Ltd. [License No. SCXK (Hu) 2022-0004], acclimatized in specific pathogen free facility under 12 h light/dark cycles. All rats had ad libitum access to standard chow and sterilized water.

Following one week of acclimation, researchers randomly divided the animals into five cohorts (10 rats per group): control (CON) group, model (MOD) group, BEN-positive control (BEN) group, and Verbenalin different doses groups [low-dose group (Verbenalin-20) and high-dose group (Verbenalin-40)]. In the study, the low- and high-dose groups received Verbenalin interventions at dosages of 20 mg/kg and 40 mg/kg, respectively.

The experimental GA model was induced through a sequential protocol commencing with daily intraperitoneal administration of PO (300 mg/kg) combined with oral HX (300 mg/kg) to MOD, BEN, and Verbenalin groups from days 1–7. MSU suspension (25 mg/L, 0.2 mL) was intraarticularly administered to the right ankle joint on day 6 (15, 16). One hour after the PO and HX treatments, the rats received daily oral administrations for 7 days as follows: the BEN group received 4.5 mg/kg BEN; the low- and high-dose Verbenalin groups received 20 mg/kg and 40 mg/kg Verbenalin, respectively. The dose for treating the rats with Verbenalin was determined in accordance with the guidelines provided by the references (17–19). At the experimental endpoint, rats were anesthetized with 3% pentobarbital sodium (intraperitoneal injection) for serum and tissue collection.

### 2.3 Evaluation of ankle joint swelling in rats

On Day 6, after the injection of MSU, the degree of swelling of rat ankles was evaluated at 4, 8, and 24 h by the foot or ankle swelling measurement, and the arthritis and gait score. The swelling rate of foot and ankle was calculated as follows: Foot or ankle swelling rate (%) =(swelling foot diameter or ankle after the injection of MSU at each timepoint/initial diameter of rat foot or ankle − 1) × 100% (20). The progression of gouty arthritis was assessed through standardized measurements of right ankle joint swelling, a hallmark pathological feature of this condition. Joint inflammation was quantitatively assessed using an established 4-point scale (0-3) to grade erythema and swelling severity, according to the method described by Coderre et al (21). Concurrently, locomotor impairment was assessed via gait analysis using a validated 4-level scoring system (1-4), with evaluation criteria based on previously published standards (21).

To ensure measurement consistency and minimize inter-observer variability, all assessments were performed by a single trained investigator throughout the study.

### 2.4 Sample collection and preparation

On 7^th^ day, the abdominal aorta of rats was punctured under anesthesia for blood testing. All blood samples were left at room temperature for 1 h and centrifuged for 15 min at 4 °C and 3500 × g. The serum was separated and then kept at -80 °C. Immediately after death of the rats, the right ankle joint was incised and cut along the median to expose the ankle joint cavity, which was preserved in 4% paraformaldehyde solution. Tissues were quickly removed on an ice rack, blotted dry on filter paper, weighed on an electronic analytical balance, and renal coefficients were calculated. A portion of the tissue was fixed with 4% paraformaldehyde and prepared for use, while the remaining tissue was stored at -80 °C. A 10% tissue homogenate was prepared by mixing tissue from each rat with cold saline solution. All homogenized samples were centrifuged at 4 °C and 3500 rpm for 10 min. Transfer the supernatant and store at -80 °C until analysis.

### 2.5 Hematoxylin and eosin staining

Cut the tissue into approximately 4-8 μm sections and dewax in xylene for 10 min. Then, transfer the sections into absolute ethanol for 5 min to remove residual xylene. Follow with three washes in PBS, each for 5 min. Immerse the sections in 120 μL of hematoxylin solution and stain for 10 min. After staining, rinse with distilled water to remove excess dye. Differentiate the sections with 1% hydrochloric acid ethanol to eliminate nuclear and cytoplasmic background staining. Subsequently, dehydrate sequentially in 80%, 95%, and absolute ethanol, each for 5 s to 2 min. After drying, mount the slides with neutral resin and observe and photograph under a microscope.

### 2.6 Measurement of biochemical kit

Serum and urinary levels of UA, CRE, and BUN were measured using commercial assay kits according to the manufacturer’s instructions. Similarly, hepatic ALT, AST, and XOD levels were determined using corresponding detection kits following standard protocols.

### 2.7 RNA-seq analysis

RNA sequencing analysis of rat kidney and liver tissues was conducted following standardized protocols. Total RNA was extracted from nine biological samples (three per group: CON, MOD, and Verbenalin-L) using TRIzol reagent (Invitrogen, CA, USA), followed by library preparation with the VAHTS Universal V6 RNA-seq Library Prep Kit. After the library construction was completed, the Qubit™ 2.0 Fluorometer was used for preliminary quantification first, diluting the library to 1.5ng/uL. Subsequently, the Agilent 2100 bioanalyzer was used to detect the insert size of the library. After the insert size met the expectations, RT-qPCR accurately quantified the effective concentration of the library (the effective concentration of the library was higher than 2 nM) to ensure the quality of the library. After the library inspection is qualified, different libraries are pooled according to the effective concentration and the target data volume required for sequencing, and 150 bp paired end readings are generated. Paired-end sequencing was performed by Wekemo Tech Group Co., Ltd (Shenzhen, China).

The image data of sequencing fragments measured by high-throughput sequencers are converted into sequence data (reads) through CASAVA base recognition. The files are in fastg format and mainly contain the sequence information of the sequencing fragments and their corresponding sequencing quality information. The raw data obtained from sequencing contains a small number of reads with sequencing headers or of low sequencing quality. To ensure the quality and reliability of data analysis, it is necessary to filter the original data. It mainly includes removing reads with adapters, reads containing N(where N indicates that the base information cannot be determined), and low-quality reads (where the number of bases with Qphred ≤20 accounts for more than 50% of the entire read length). All subsequent analyses are high-quality ones based on cleandata. For data analysis, the reference genome and annotations were retrieved from NCBI. Sequence alignment was performed using HISAT2 (v2.0.5) after building the genome index. Gene expression levels were quantified in FPKM values through StringTie analysis. Differential expression analysis was conducted using DESeq2 (v1.16.1) with a significance threshold of adjusted *p*-value < 0.05 after Benjamini-Hochberg correction. Functional enrichment analyses included Gene Ontology (GO) and KEGG pathway analyses using the cluster Profiler package, with corrected *p*-value < 0.05 considered statistically significant.

### 2.8 Metagenomic analysis

After the last administration, fresh cecum contents from rats were collected in sterile tubes and stored at -80 °C until gut microbiota analysis. Total genomic DNA of the microbial community genomic was extracted from cecum contents using the E.Z.N.A.^®^ soil DNA Kit (Omega Bio-tek, Norcross, GA, U.S.) according to manufacturer’s instructions. Amplicon sequencing was performed using the Illumina MiSeq PE300 platform/NovaSeq PE250 platform (Illumina, San Diego, CA, USA) according to the standard protocols of Wekemo Tech Group Co., Ltd. (Shenzhen China).

To ensure the reliability of the data, it is necessary to use Kncaddata software to preprocess the Raw sequencing data. First, remove the joint sequences from the original data and the sequences with low quality (default quality threshold ≤20), as well as the sequences with a final length of less than 50bp. Considering that the samples may have host contamination, it is necessary to align the Clean Data to the host genome. By default, the Bowwtie2 software is used to filter the sequences from the host and obtain valid sequences for subsequent analysis. Finally, the rationality and effectiveness of quality control were tested through FastOC. The sequence number of species contained in the samples was calculated by comparing Kraken2 with the self-built microbial nucleic acid database, and then Bracken was used to predict the actual relative abundance of species in the samples. The variation in the gut microbiota among the CON, MOD, and Verbenalin-L groups was assessed via non-metric multidimensional scaling (NMDS) analysis. Linear discriminant analysis coupled with effect size (LEfSe) was performed to identify the dominant microbiota from phylum to genus, according to a standard LDA score of >2, *p* < 0.05 in different groups.

### 2.9 Metabolomics analysis

The urine samples (100 μL) were placed in the EP tubes and resuspended with prechilled 80% methanol by well vortex. Then the samples were incubated on ice for 5 min and centrifuged at 15000 g, 4 °C for 20 min. Some of supernatant was diluted to final concentration containing 53% methanol by LC-MS grade water. The samples were subsequently transferred to a fresh Eppendorf tube and then were centrifuged at 15000 g, 4 °C for 20 min. Finally, the supernatant was injected into the LC-MS/MS system analysis.

LC-MS analyses were performed using a Vanquish UHPLC system (Thermo Fisher, USA) coupled with an Orbitrap Q ExactiveTM HF mass spectrometer or Orbitrap Q Exactive TMHF-X mass spectrometer (Thermo Fisher, Germany). Samples were injected onto a Hypersil Gold column (100 mm × 2.1 mm, 1.9 μm) using a 12-min linear gradient at a flow rate of 0.2 mL/min. The eluents for the positive and negative polarity modes were eluent A (0.1% formic acid aqueous solution) and eluent B (methanol). The solvent gradient was set as follows: 0-1.5 min, 2% B; 1.5–3 min, 2%-85% B; 3–10 min, 85%-100% B; 10-10.1 min, 100%-2% B; 10.1–12 min, 2% B. Q ExactiveTM HF mass spectrometer was operated in positive/negative polarity mode with spray voltage of 3.5 kV, capillary temperature of 320 °C, sheath gas flow rate of 35 psi and aux gas flow rate of 10 L/min, S-lens RF level of 60, Aux gas heater temperature of 350 °C.

The offline data files are converted into mzXML format through ProteoWizard. First, XCMS is used for peak extraction and peak quantification. Through parameters such as retention time and mass-to-charge ratio, peak alignment is performed for different samples, and the peak area of the first sample is corrected to make the quantification more accurate. Then, based on the information such as the 10ppm mass deviation and the added ions set, it is compared with the high-quality secondary spectrum database for metabolite identification. Subsequently, the blank sample was used to remove background ions. The original quantitative results were standardized according to the formula: original quantitative value of the sample/(total quantitative values of sample metabolites/total quantitative values of the first sample metabolites), and the relative peak area was obtained. These metabolites were annotated using the KEGG database (https://www.genome.jp/kegg/pathway.html), HMDB database (https://hmdb.ca/metabolites) and LIPID Maps database (http://www.lipidmaps.org/).

### 2.10 Western blot assay

Kidney and liver tissue samples were homogenized in 200 μL RIPA lysis buffer supplemented with 1% protein phosphatase inhibitor cocktail. After centrifugation at 12,000 × g for 15 min at 4 °C, the supernatant was collected for protein quantification using a BCA assay kit. Protein concentrations were normalized, and samples were mixed with 5× loading buffer (20% of final volume). Following denaturation at 100 °C for 10 min, proteins were separated by SDS-PAGE and transferred onto PVDF membranes. The membranes were blocked with 5% skim milk in TBST for 1.5 h at room temperature. Following blocking, the membranes were incubated overnight at 4 °C with primary antibodies targeting GLUT9 (1:1000), URAT1 (1:1000), XOD (1:1000), p-PI3K (1:1000), PI3K (1:1000), p-AKT (1:1000), AKT (1:1000), p-MAPK (1:1000), MAPK (1:1000), NLRP3 (1:1000), IL-1β (1:1000), β-actin (1:5000), respectively. β-actin was served as loading control for normalization of protein content across all immunoblot analyses. After three TBST washes, membranes were probed with species-matched HRP-conjugated secondary antibodies (1:5000 dilution; goat anti-rabbit or goat anti-mouse) for 1.5 h at room temperature. Following additional TBST washes, protein bands were visualized using an ECL substrate (1:1 mixture of components A and B) and imaged with an Amersham Image Quant 800 system. Band intensities were quantified using Image Lab software.

### 2.11 Statistical analysis

All statistical analyses were performed using the GraphPad Prism software 8.0 program. Measurements were expressed as mean ± standard deviation and the repeatability of each experiment was greater than or equal to 3. One-way analysis of variance (ANOVA) was used to compare normally distributed data between multiple groups and repeated measures and two-way ANOVA were used to analyze ankle swelling rate, foot swelling rate, inflammation scores, and gait scores, with *p* < 0.05 being considered statistically significant.

## 3 Results

### 3.1 Verbenalin ameliorates joint inflammation and pathological changes in a rat model of gout

**Figure 1:**
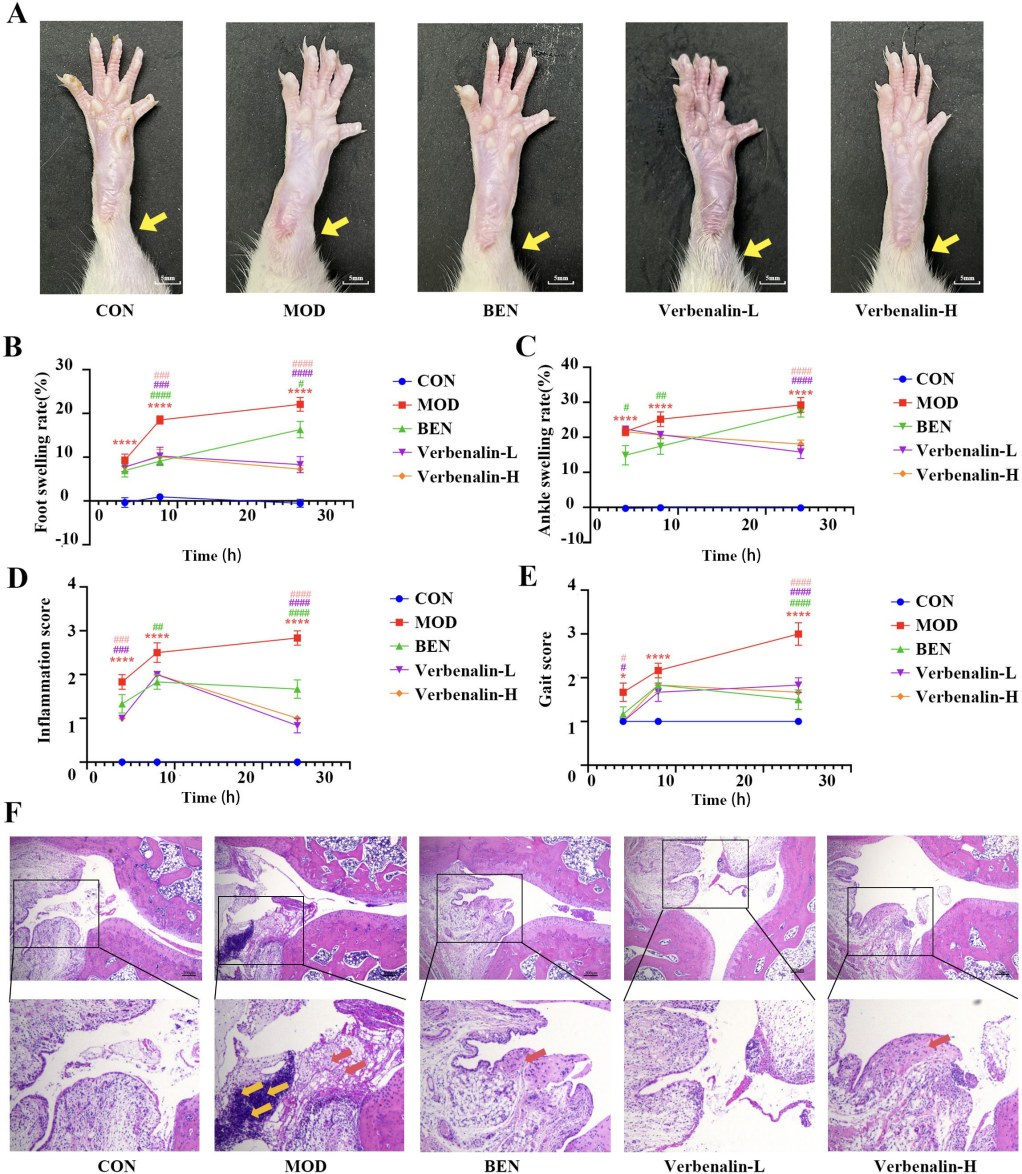
Verbenalin alleviated joint pathology in gout. **(A)** picture of the right ankle joint of rats 24 h after MSU suspension injection (n=6); **(B)** foot swelling rate (n=6). **(C)** ankle swelling rate (n=6); **(D)** inflammation score (n=6); **(E)** gait score (n=6); **(F)** H&E staining of the ankle joint (scale bar =50μm, magnification ×200, ×400). ^*^p < 0.05, ^****^p < 0.0001 *vs*. CON group; ^#^*p* < 0.05, ^##^*p* < 0.01, ^###^*p* < 0.001, ^####^*p* < 0.0001, *vs*. MOD group. The red arrow indicates synovial hyperplasia tissue, while the yellow arrow indicates inflammatory cell infiltration.

Following intra-articular MSU injection, the right ankle joints exhibited marked swelling (**Figure 1A**). The model group increased significantly in foot swelling and toe volume compared to controls, reaching the highest level within the observation period at the 24th hour, confirming successful model establishment (**Figures 1B, C**). Verbenalin treatment significantly attenuated both ankle and foot edema at all observed time points (4, 8, and 24 h). Concurrently, it reduced inflammation scores (**Figure 1D**) and gait scores (**Figure 1E**), demonstrating improved joint inflammation.

Histopathological analysis of H&E staining ankle joint sections revealed distinct morphological changes among groups (**Figure 1F**). Control animals displayed normal synovial morphology with well-organized cell layers and absent inflammatory infiltrates. Model group specimens exhibited characteristic gout-associated pathology including synovial hyperplasia (increased cell layers), disrupted tissue architecture, and dense inflammatory cell infiltration. Verbenalin treatment groups showed significant mitigation of these pathological features, with dose-dependent reductions in inflammatory infiltration and restoration of synovial tissue organization. These histological findings confirm verbenalin’s therapeutic potential in alleviating MSU-induced joint pathology in this gout model.

### 3.2 Verbenalin ameliorates renal dysfunction and histopathological damage in a rat model of gout

**Figure 2:**
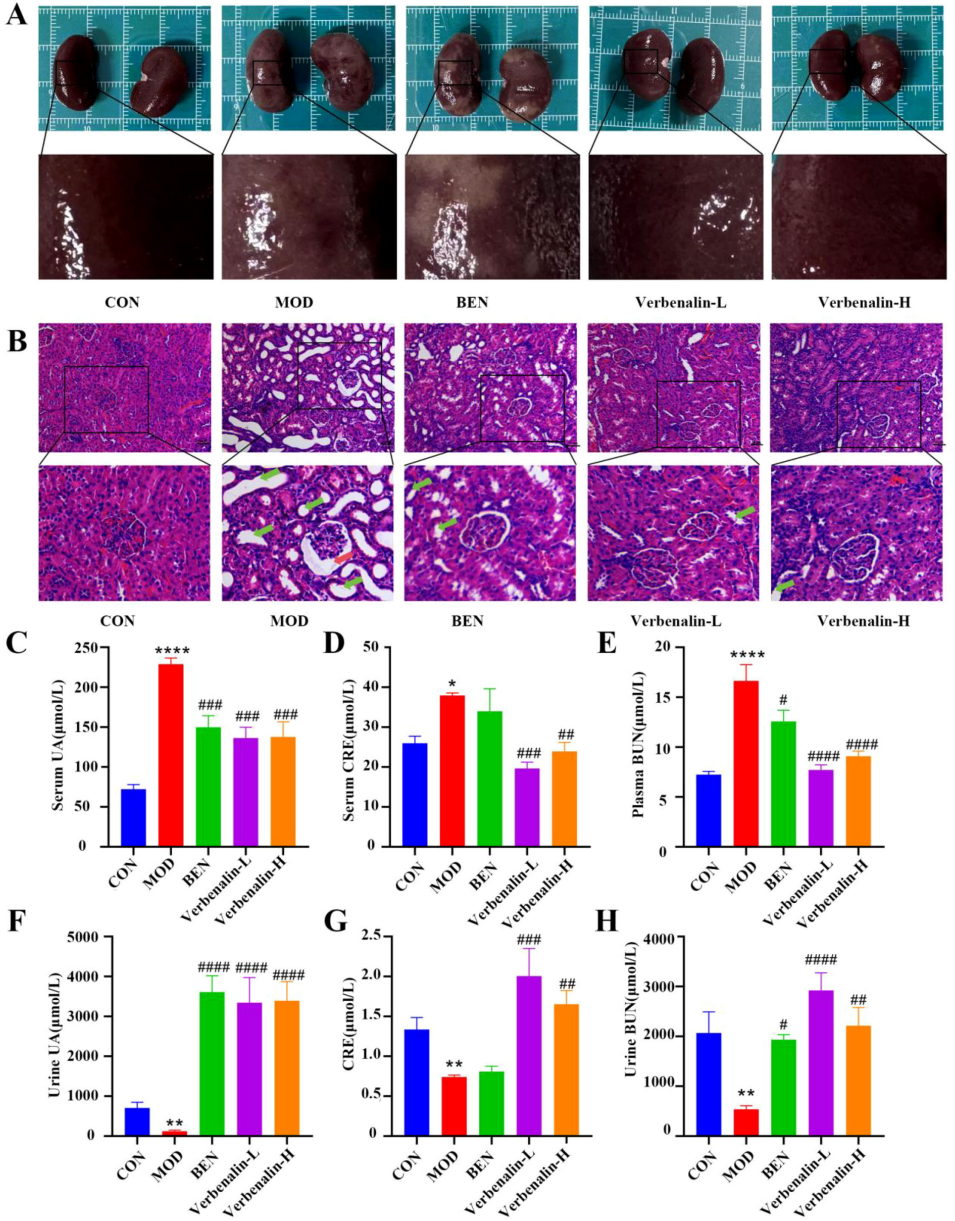
Verbenalin reduced kidney damage and promoted UA excretion. **(A)** picture of rat kidney (n=6); **(B)** renal H&E staining (scale bar =50μm, magnification ×200, ×400). The red arrow indicates dysplasia of the renal capsule, while the green arrow indicates renal tubular vacuolar degeneration. **(C)** serum UA (n=6); **(D)** serum CRE (n=6); **(E)** plasma BUN (n=6); **(F)** urine UA (n=6); **(G)** CRE clearance (n=6); **(H)** urine BUN (n=6). ^*^p < 0.05, ^**^p < 0.01, ^****^p < 0.0001 *vs*. CON group; ^#^*p* < 0.05, ^##^*p* < 0.01, ^###^*p* < 0.001, ^####^*p* < 0.0001 *vs*. MOD group.

In the experiment, we assessed how verbenalin affected the liver and kidney damage in gout-induced rats. Rats in the control group had smooth, vermilion kidneys, as seen in **Figures 2A, B**. The kidney tissue structure was normal, with clear tubules and glomeruli, a regular lumen, and a well-organized cellular structure. In the model group, the kidneys were enlarged in size, the surface was rough, and white lesions could be seen. There were obvious inflammatory cell infiltration in the kidney tissue, and the renal tubules were disorganized with coarse vacuolated degeneration. The Baumann’s capsule that encapsulated the glomeruli was dilated, and the glomeruli inside the capsule cavity were not filled. Compared with the model group, the kidney surface of rats in the verbenalin group was smooth, the white lesion area was reduced, and the degree of renal tissue lesions were all reduced to a certain extent.

Meanwhile, we detected UA level of blood and urine, a key indicator of gout, as well as BUN and CRE, an indicator of renal function, to evaluate the pharmacodynamic effect of anti-gout. Compared with the control group, the model group showed a significant increase in blood UA, urea nitrogen and CRE content (**Figures 2C–E**), and a significant decrease in urinary UA, urea nitrogen and CRE clearance (**Figures 2F–H**), whereas verbenalin treatment effectively promoted UA excretion, reduced serum UA content and improved renal function in gout-induced rats, with the effect of the low-dose group of verbenalin being slightly superior to that of the high-dose group.

### 3.3 Verbenalin improves hepatic function and mitigates histopathological changes in a rat model of gout

**Figure 3:**
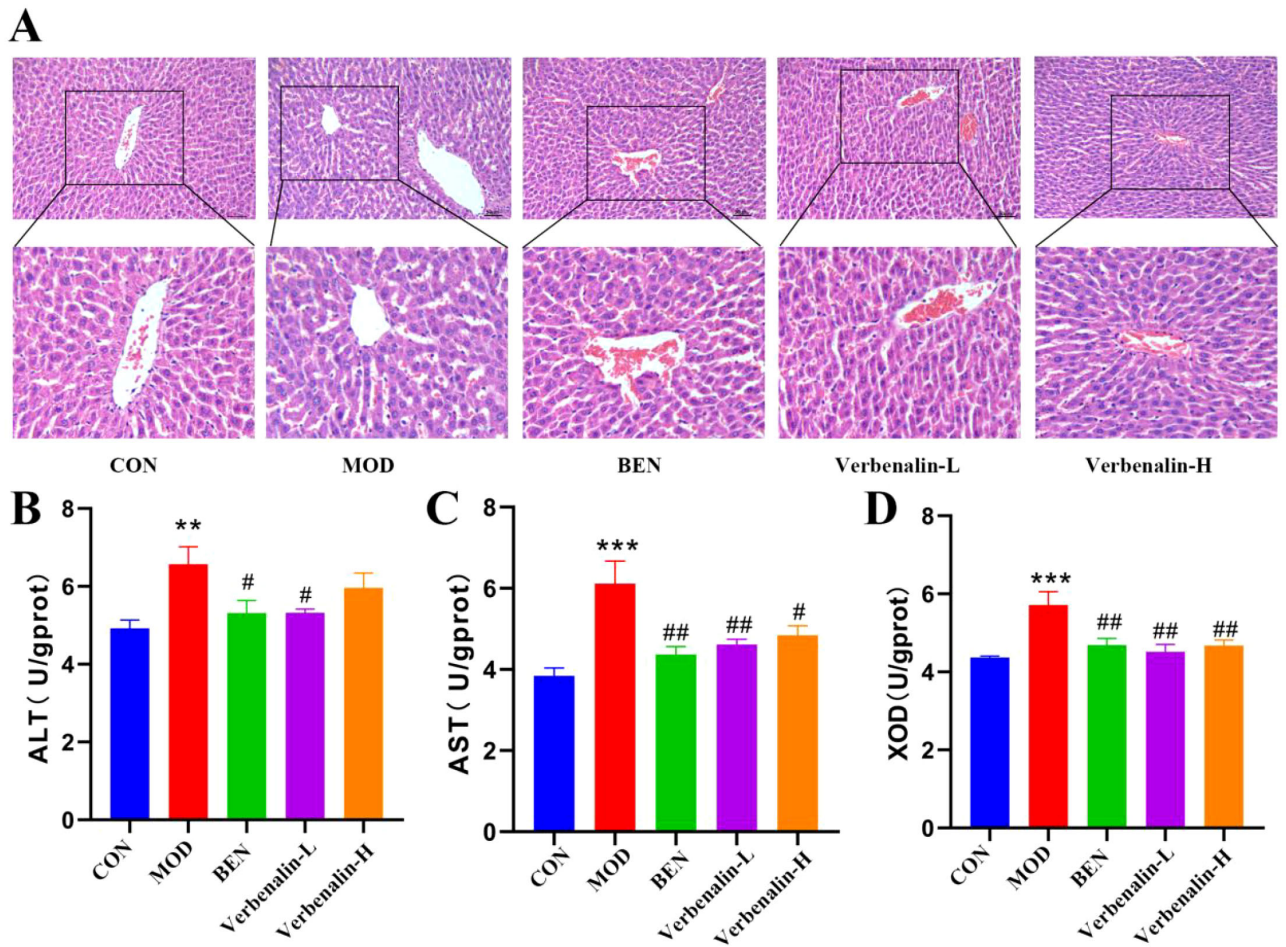
Verbenalin mitigated hepatic injury and suppressed UA synthesis. **(A)** rat hepatic H&E staining (scale bar =50μm, magnification ×200, ×400); **(B)** Liver ALT (n=6); **(C)** Liver AST (n=6); **(D)** Liver XOD (n=6). ^**^p < 0.01, ^***^p < 0.001 *vs*. CON group; ^#^*p* < 0.05, ^##^*p* < 0.01 *vs*. MOD group.

As shown in **Figure 3A**, the liver tissues of rats in the control group were normal in structure, and the nuclei of liver cells were clear, tightly and neatly arranged. In the model group, the cytoplasm was loose and lightly stained. Compared with the model group, the degree of liver tissue lesions in the verbenalin group was reduced to a certain extent. Verbenalin could significantly reduce the increase in liver AST and ALT levels caused by the gout model, and improve the liver injury in rats (**Figures 3B, C**). The results showed that the expression of XOD(**Figure 3D**), a key enzyme related to purine production, was significantly up-regulated in the model group, while the expression was reversed in the verbenalin group, suggesting that verbenalin may reduce UA production by inhibiting the activity of key enzymes related to purine production. According to the biochemical indexes and H&E staining results, we found that verbenalin has a certain protective effect on liver injury caused by gout, and can inhibit liver UA synthesis. Low dose verbenalin is more effective than high dose verbenalin.

As low-dose verbenalin has demonstrated sufficient efficacy in reducing UA and key inflammatory indicators, and is slightly better than the high-dose group, in order to explore the specific mechanism of its therapeutic effect, this group was selected for subsequent omics analysis.

### 3.4 Transcriptomics with western validation reveals verbenalin ameliorates gouty renal injury through PI3K/AKT inhibition

**Figure 4:**
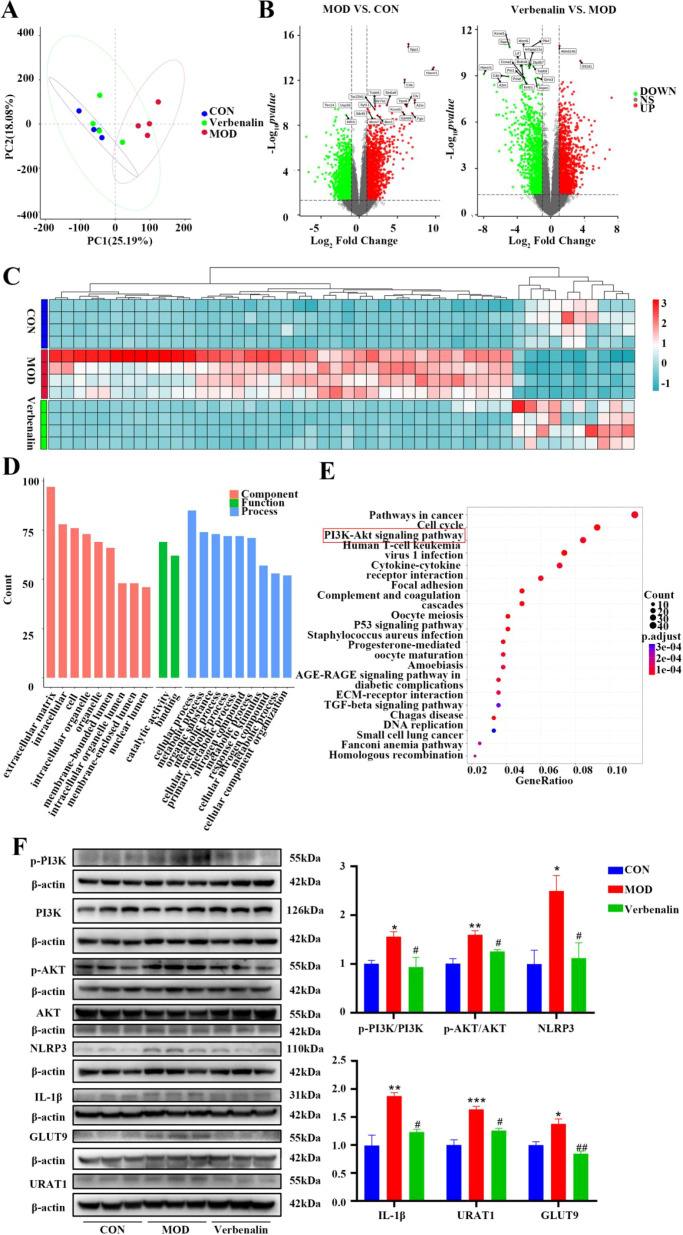
Verbenalin inhibited the renal PI3K-AKT signaling pathway (n=4). **(A)** PCA plot; **(B)** volcano plot; **(C)** heatmap of top 50 genes of difference; **(D)** GO analysis; **(E)** KEGG analysis; **(F)** renal PI3K-AKT signaling pathway related protein (n=3). ^*^p < 0.05, ^**^p < 0.01, ^***^p < 0.001 *vs*. CON group; ^#^*p* < 0.05, ^##^*p* < 0.01 *vs*. MOD group.

Transcriptomics analysis was performed on rat kidneys, compared with the control group, the samples from the model and verbenalin groups were able to separate significantly, indicating that there were significant differences in genes between the groups (**Figure 4A**). Compared with the control group, 2416 DEGs were identified in the model group, of which 1243 DEGs were up-regulated and 1173 DEGs were down-regulated, and 2472 DEGs were identified in the verbenalin group, of which 1283 DEGs were up-regulated and 1189 DEGs were down-regulated, compared with the model group (**Figure 4B**). Heat map clustering analysis (**Figure 4C**) showed that these differentially expressed mRNAs could well distinguish the verbenalin group from the model group.

GO enrichment analysis (**Figure 4D**) was performed on the differential genes, which were mainly distributed in the extracellular matrix, intracellular and other cellular components. Through catalytic activity, binding and other mechanisms, they are involved in cellular processes, organic metabolic processes, etc. KEGG pathway analysis (**Figure 4E**) revealed that cancer pathway, cell cycle, and PI3K-AKT signaling pathways were significantly enriched. Among them, PI3K-AKT signaling pathway is an important autophagy and inflammation-related signaling pathway, which is closely related to the biological processes of UA and lipid metabolism, and can affect the expression of (URAT1) and (GLUT9) (22, 23). Verbenalin may exert its therapeutic effect on the gout model by affecting the PI3K-AKT signaling pathway. therapeutic effects. Therefore, WB validation of key proteins of the PI3K-AKT signaling pathway was performed, and it was found that verbenalin significantly inhibited the expression of PI3K-AKT signaling pathway proteins and significantly reduced the expression of URAT1 and GLUT9 (**Figure 4F**). Verbenalin may inhibit renal inflammation, reduce UA reabsorption, and promote UA excretion by inhibiting the renal PI3K-AKT signaling pathway.

### 3.5 Transcriptomics with western validation reveals verbenalin inhibits the MAPK signaling pathway in the liver

**Figure 5:**
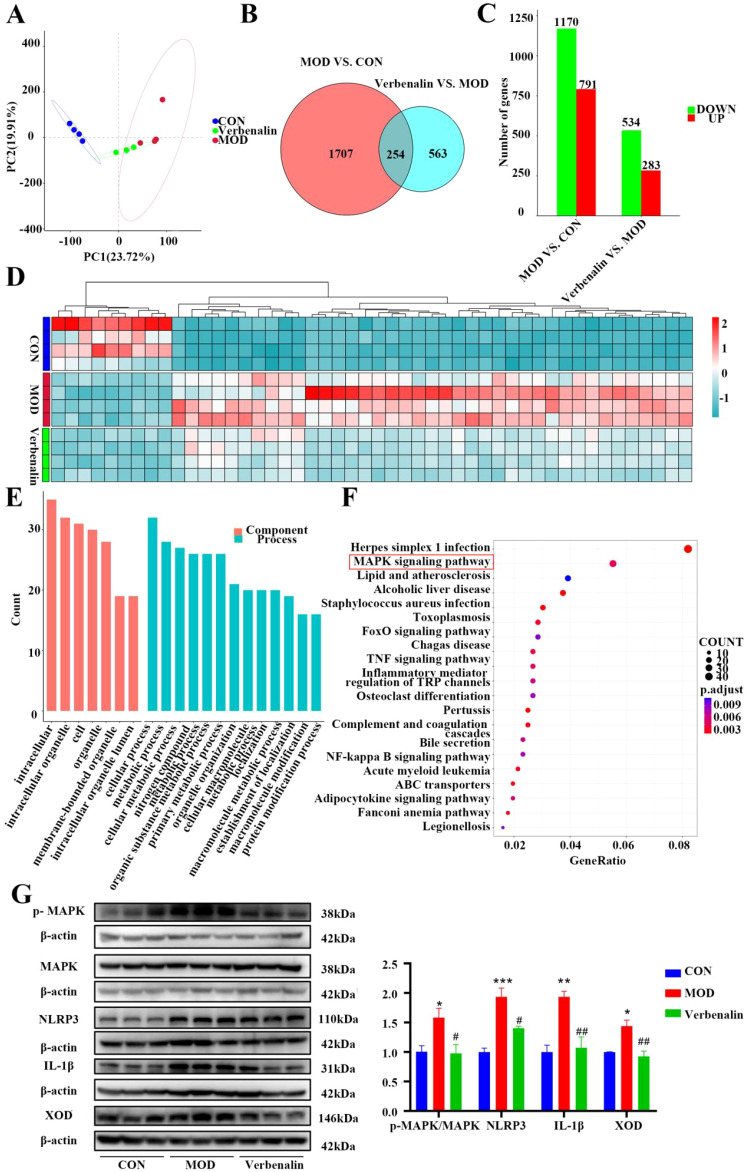
Verbenalin inhibited the liver MAPK signaling pathway (n=4). **(A)** PCA plot; **(B)** Venn diagram; **(C)** differential gene cylinder; **(D)** heatmap of top 50 genes of difference; **(E)** GO analysis; **(F)** KEGG analysis; **(G)** hepatic MAPK signaling pathway related protein (n=3). ^*^p < 0.05, ^**^p < 0.01, ^***^p < 0.001 *vs*. CON group; ^#^*p* < 0.05, ^##^*p* < 0.01 *vs*. MOD group.

Transcriptomics analysis was performed on rat liver, compared with the control group, the samples in the model and verbenalin groups were able to separate significantly, indicating that there were significant differences in genes between the groups (**Figure 5A**). Compared with the control group, 1961 DEGs were identified in the model group, of which 791 DEGs were up-regulated and 1170 DEGs were down-regulated; 817 DEGs were identified in the verbenalin group, of which 283 DEGs were up-regulated and 534 DEGs were down-regulated, compared with the model group (**Figures 5B, C**). Heat map clustering analysis (**Figure 5D**) showed that these differentially expressed mRNAs could well distinguish the verbenalin group from the model group.

GO enrichment analysis (**Figure 5E**) was performed on the differential genes, which were mainly distributed in intracellular components such as intracellular and intracellular organelles, and were involved in cellular processes, metabolic processes, and so on. KEGG pathway analysis (**Figure 5F**) revealed that herpes simplex virus infection, MAPK signaling pathway, lipids and atherosclerosis were significantly enriched. UA is produced in the liver via purine metabolism, and XOD plays a key role in purine metabolism, catalyzing the oxidation of HX to xanthine, and then to UA (24). The MAPK signaling pathway is one of the most important pathways that are known to be closely related to the pathogenesis of gout, and it has been demonstrated that phosphorylation of MAPK promotes the expression of XOD (25). Therefore, WB validation of key proteins of the MAPK signaling pathway was performed, and verbenalin was found to significantly inhibit the expression of MAPK signaling pathway proteins and significantly reduce the expression of XOD (**Figure 5G**). Verbenalin may inhibit hepatic inflammation and reduce UA production by inhibiting the hepatic MAPK signaling pathway.

### 3.6 Metagenomic analysis reveals Verbenalin modulates gout-associated gut microbiota

**Figure 6:**
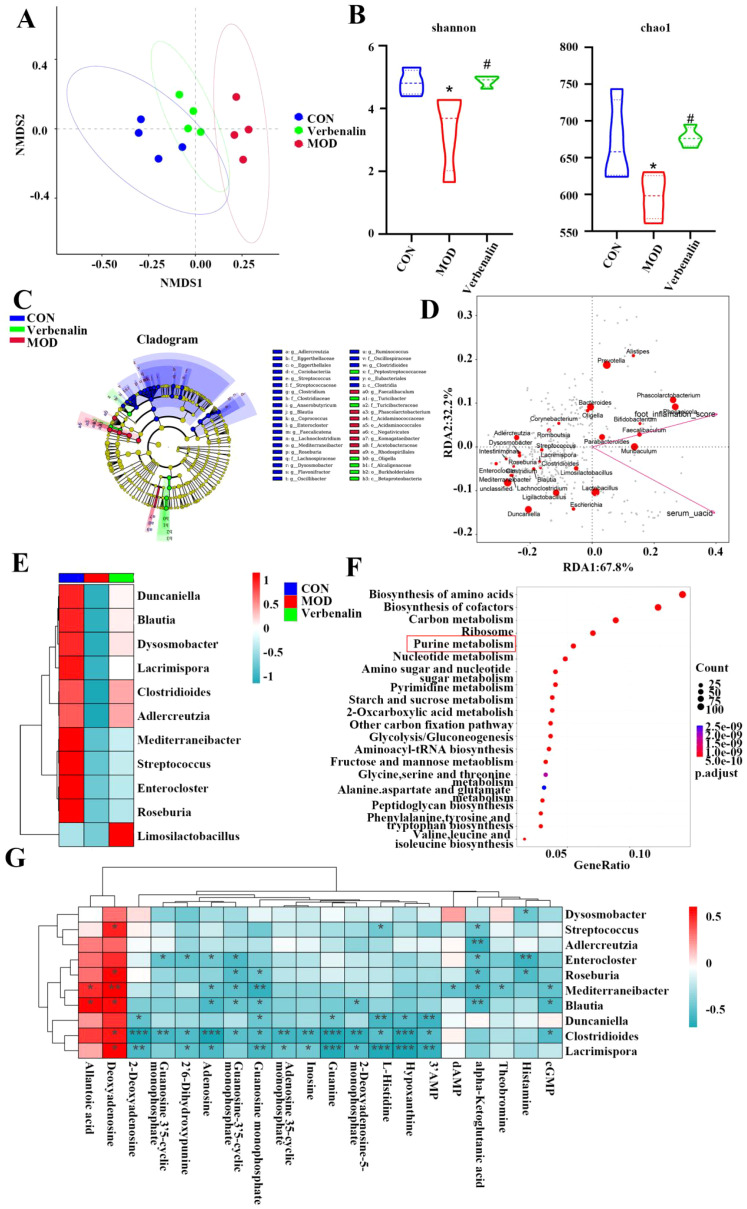
Verbenalin modulated the gut microbiota associated with gout (n=4). **(A)** PCA plot; **(B)** α-diversity analysis; **(C)** LEfSe analysis; **(D)** RDA analysis plot; **(E)** Heat map of gout associated flora; **(F)** KEGG pathway analysis; **(G)** Correlation analysis diagram of gout associated flora and purine metabolites. ^*^p < 0.05 *vs*. CON group; ^#^*p* < 0.05 *vs*. MOD group.

Metagenomic analysis of rat feces and PCA results showed significant differences in fecal flora between the control, model and verbenalin groups, with samples from the control and model groups being able to be significantly separated, and samples from the verbenalin group being more similar to the control group (**Figure 6A**). Compared with the control group, the model group showed a decrease in the alpha diversity of the flora, with a significant decrease in the shannon index and chao1 index, and a significant increase in the alpha diversity of the flora in the verbenalin group, suggesting that verbenalin significantly improved the richness of the intestinal flora species in gout-induced rats (**Figure 6B**). Intergroup comparisons among the 3 subgroups were performed using LEfSe analysis, and the results are shown in **Figure 6C**, which showed that the microbial communities of the 3 groups differed, with 25, 8, and 7 dominant taxa screened in the control, model, and verbenalin groups, respectively. Among these taxa, *Lachnospiraceae*, *Eggerthellaceae*, and *Oscillospiraceae* were significantly more abundant in the control group, while *Acidaminococcaceae* and *Acetobacteraceae* were more abundant in the model group. In contrast, *Peptostreptococcaceae*, *Alcaligenaceae* were more abundant in the verbenalin group. These phylogenetic subtypes are essential for distinguishing the composition of the gut microbiota in the three groups. In summary, verbenalin improved the gut microbiota structure of gout-induced rats to some extent.

RDA analysis showed that ankle joint swelling was positively correlated with blood UA level in rats, and ankle joint swelling and blood UA level were significantly and positively correlated with the levels of *Phascolarctobacterium*, *Phocaeicola*, *Parabacteroides*, etc., and with *Intestinimonas*, *Mediterraneibacter, Enterocloster*, etc. were significantly negatively correlated (**Figure 6D**). Therefore, we made heat maps of the contents of gout associated bacteria in the three groups (**Figure 6E**). The results showed that compared with the control group, the abundance of gout negatively associated bacteria, such as *Duncaniella*, *Blautia* and *Dysosmobacter*, decreased in the model group, and Verbenalin treatment could increase the abundance of these beneficial bacteria. KEGG pathway enrichment analysis showed that the differential genes were mainly enriched in amino acid biosynthesis, cofactor biosynthesis synthesis, and carbon metabolism pathways, while purine metabolism pathway was significantly enriched (**Figure 6F**). Purine metabolism disorder is an important cause of abnormal UA level in the body. We conducted correlation analysis between these gout related flora and purine metabolites (**Figure 6G**), and the correlation analysis results showed that the abundance of these flora was significantly correlated with the content of purine metabolites, and gout related flora could significantly regulate purine metabolism. It was hypothesized that Verbenalin may regulate intestinal metabolism and improve UA metabolism disorder by regulating the level of intestinal.

### 3.7 Untargeted urine metabolomics reveals Verbenalin regulates purine metabolites in gout model rats

**Figure 7:**
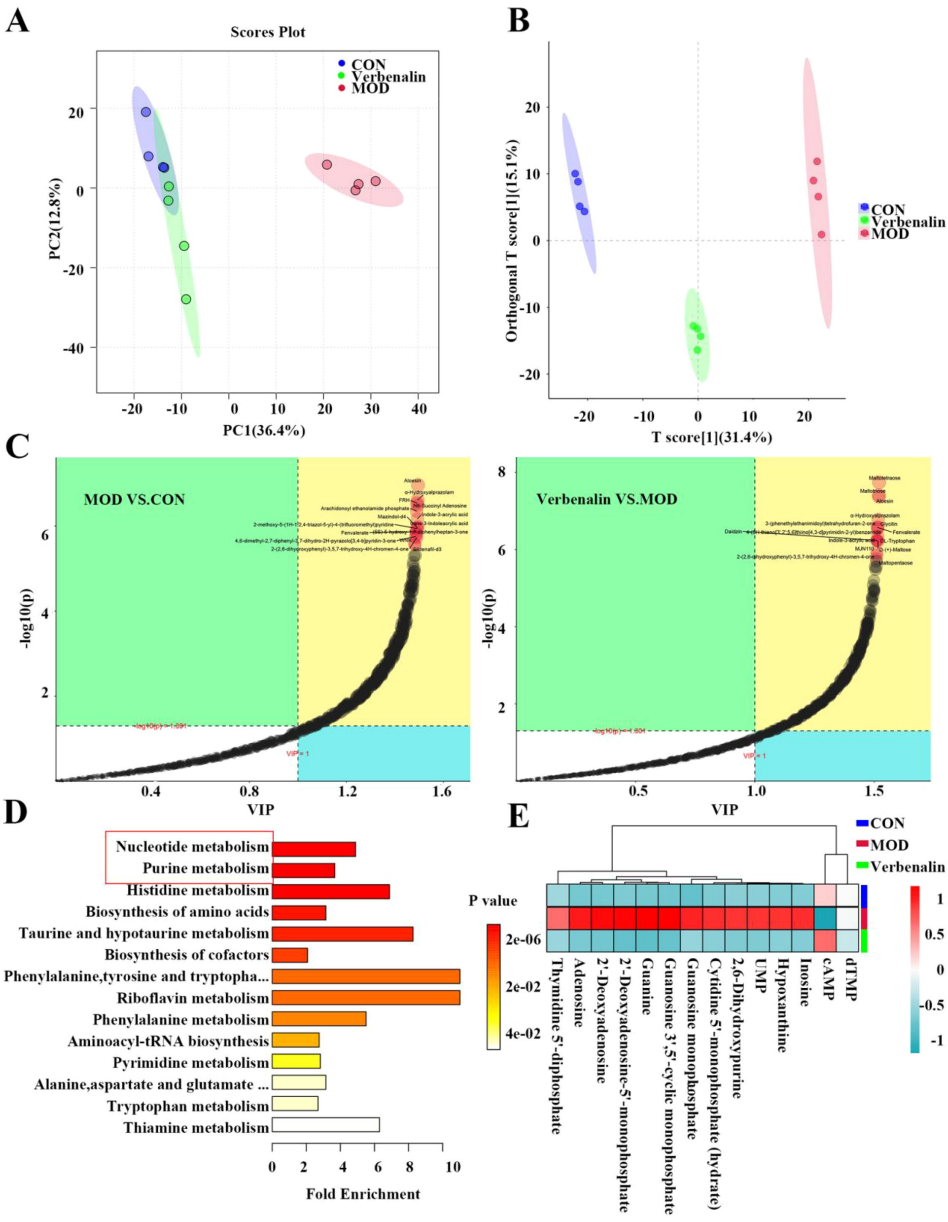
Verbenalin regulated purine metabolites. **(A)** PCA plot; **(B)** OPLS-DA plot; **(C)** MOD vs. CON VIP plot, Verbenalin vs MOD. VIP plot; **(D)** KEGG plot; **(E)** heat map of purine metabolites.

Untargeted metabolomics analysis was used to study the differences in urinary metabolites between the control, model and verbenalin groups. According to the unsupervised multivariate analysis method PCA, the metabolites of all three groups were significantly different, especially in the control and model groups, and the samples of the verbenalin group were more similar to those of the control group, which indicated that verbenalin could improve the metabolic disorders in gout-induced rats (**Figure 7A**). Metabolites with significant differences were selected based on the VIP obtained from the OPLS-DA model and the p-value of the t-test, and metabolites with VIP>1 and *p* < 0.05 were significantly different (**Figure 7B**). Among these metabolites, 477 differential metabolites were obtained in relation to the model group compared to the control group, including Aloesin, trans-3-Indoleacrylic acid, and pseudo-Hydroxyalprazolam. in addition, 462 differential metabolites were identified between the verbenalin group and the model group, including Maltotetraose, Aloesin, and Maltotriose. Notably, this differential metabolite returned to normal levels and was greatly affected after verbenalin treatment (**Figure 7C**).

In order to explore the possible relevant metabolic pathways for verbenalin treatment of gout, the differential metabolites screened by OPLS-DA were imported into the metabolic analysis database for KEGG pathway analysis, which screened 14 significantly enriched significant metabolic pathways, and nucleotide metabolism and purine metabolism were the most significantly enriched differential metabolic pathways, suggesting that verbenalin may decrease the level of purine metabolism *in vivo* by modulating the level of UA production (**Figure 7D**). HUA caused by purine nucleotide metabolism disorders is the main cause of gout development (2). Therefore, we calculated the urine purine metabolity-related metabolites content and made a heat map (**Figure 7E**). The results showed that there were significant differences in purinine-related metabolites between the model group and the blank group, and verbenalin reversed this change. It is suggested that verbenalin may reduce UA by regulating purine metabolism.

## 4 Discussion

Huazhuo Sanjie Chubi Decoction is an empirical formula that is based on the understanding of the pathogenesis of gout and its symptomatic characteristics and the pathogenesis of “spleen deficiency, dampness, phlegm, stagnation, heat and stagnation”, and is summarized and condensed, with remarkable efficacy in the clinical treatment of gout (26). Through quantitative and cellular verification of the components of Huazhuo Sanjie Chubi Decoction, the research group found that verbenalin has a good potential for the treatment of gout, but its therapeutic effect and effect on gout have not been fully explained. We used a rat model of HUA combined with acute gouty arthritis in order to assess the effectiveness of verbenalin. This model can more accurately depict the entire pathological process in gout patients, from metabolic abnormality to acute inflammatory attack (27). Histological analysis revealed synovial tissue hyperplasia with a high number of neutrophil infiltration and a significant increase in blood UA in the joint tissues of the model group, demonstrating that the model construction was successful. The results demonstrated that MSU crystal injection caused joint swelling, increased inflammation, and improved gait scores in rats. Additionally, gout-induced rats respond well to verbenalin treatment, which can effectively lower serum UA levels, promote urine UA excretion, block the infiltration of inflammatory cells into the synovium and joint cavity, and reduce joint swelling to a level that is almost normal.

The direct cause and biochemical basis of gout is HUA, and when serum UA concentration exceeds 6.5 mg/dL, serum sodium urate crystals are oversaturated with MSU crystals, and MSU crystal deposition in the organs leads to a series of inflammatory responses and organ damage (28). Deposition of MSU crystals in organs activates natural immune responses such as proliferation and polarization of macrophages, monocytes, and neutrophils, as well as release of inflammatory factors with induced activation of inflammatory vesicles, resulting in an inflammatory cascade response and organ damage (29). Cellular inflammatory responses dominated by NLRP3 play an important role in the course of gout, and activation of NLRP3 inflammatory vesicles and release of large amounts of IL-1β are central to MSU-mediated gouty attacks (30). Liver and kidney injury is a common complication of gout, the kidneys are the main place of UA excretion, the liver is the main place of purine metabolism and UA synthesis, liver and kidney injury will not only lead to purine metabolism disorders *in vivo*, but also caused by the degradation of DNA provides a large number of purine precursors to promote UA synthesis, which in turn further leads to HUA and gouty episodes (31–33). Therefore, we also paid attention to the effect of the gout model on the kidneys, and found that the urea nitrogen and CRE levels were abnormal in the model group; the kidneys were obviously enlarged, the tissues appeared to be lesions, and the oxidative stress levels were abnormal. At the same time, we found that the liver tissue of the model group showed lesions, and the ALT and AST levels of the liver were significantly higher than those of the control group, indicating that the gout model caused liver and kidney damage, and the administration of verbenalin effectively improved the renal function abnormality and the lesions of the kidneys and liver tissue of the model rats, and lowered the oxidative stress level of the kidneys and the transaminase level of the liver, which indicated that the verbenalin had a protective effect on the liver and kidney damage caused by gout.

The renal transcriptome results suggest to us that verbenalin may affect the kidney may be related to the PI3K-AKT signaling pathway, which is an important autophagy, inflammation-related signaling pathway. It has been shown that the PI3K-AKT signaling pathway mediates UA excretion by a mechanism related to the regulation of URAT1 and GLUT9 UA transporter proteins (34). UA excretion in the kidney mainly relies on UA transporter proteins, of which URAT1 and GLUT9 are important carriers in the process of UA reabsorption into the bloodstream. URAT1 is located in the apical membrane of proximal tubular epithelial cells, which is an important factor in the maintenance of UA levels, and it is mainly responsible for recognizing and transporter of organic anions that mediate the transfer of urate, and it can be used for the transfer of urate by the proximal tubular cells through transmembrane potential gradient. The apical membrane of tubular cells is responsible for recognizing and transporting organic anions that mediate the transport of urate from the proximal tubule lumen through a transmembrane potential gradient (35). URAT1 has a strong transport capacity and approximately 90% of UA reabsorption in the body is mediated by URAT1 (36). GLUT9, an important transporter promoting urate reabsorption, is located in the outer basement membrane of the proximal renal tubule and is mainly expressed in the kidney, liver, placenta and cartilage (37). The results showed that verbenalin may have a “synergistic impairment” effect by inhibiting the PI3K-AKT signaling pathway: on the one hand, it reduces uric acid reabsorption and improves hyperuric acid status by reducing the expression of URAT1 and GLUT9; On the other hand, by inhibiting the inflammatory response of NLRP3, it alleviates renal pathology to protect kidney function and promote UA excretion.

The MAPK pathway is significant in regulating growth signaling and is involved in cell proliferation, differentiation, apoptosis, inflammation, and immunity by phosphorylating key protein targets to cascade the activation of MAPK kinase, MAPK kinase, and MAPK to transmit extracellular signals to the nucleus, and subsequently regulating transcription of downstream associated factors (38). The MAPK signaling pathway promotes XOD expression, and use of the MAPK inhibitor can eliminate the increased XOD expression (25, 39). UA is converted from purine nucleosides and purines in the presence of XOD, which is a key catalytic enzyme for UA production (40). The experimental results of this study showed that verbenalin inhibited the MAPK signaling axis, reduced XOD-mediated UA production, and alleviated inflammation induced by a high UA environment, achieving the dual effects of “inhibiting enzyme inhibition of UA production” and “anti-inflammatory liver protection”.

Dysbiosis of the gut flora has been found to be strongly associated with numerous metabolic diseases, especially gout, and the gut microbiota is thought to influence the development of gout and serum urate levels (41). Dysbiosis of gut flora can be used as a non-invasive diagnostic marker for gout (42). The microflora diversity index chao1 and shannon in the model group were significantly lower than those in the control group, while the microflora diversity was restored after verbenalin treatment, and verbenalin could improve intestinal flora disturbance caused by gout. The results of RDA and microflora abundance heat map showed that verbenalin could reverse some changes in the abundance of gout associated microflora. It has been reported in the literature that the abundance of beneficial bacteria such as *Adlercreutzia*, *Streptococcus*, *Blautia*, *Roseburia* is significantly reduced in gout related diseases, and verbenalin can increase the abundance of these beneficial bacteria (43–46). *Streptococcus* is an important UA depressant for intestinal health and can promote UA degradation (47). *Blautia*, *Dysosmobacter* and *Mediterraneibacter* are butyric acid-producing bacteria, which produce butyric acid that maintains intestinal integrity, enhances the damaged intestinal barrier function, and can inhibit the formation of HUA by reducing the level of inflammatory factors (48, 49). Butyric acid is a short-chain fatty acids (SCFAs), and SCFAs produced by the metabolism of intestinal flora can alleviate intestinal inflammation and maintain the intestinal barrier (50). The results of KEGG enrichment showed that many genes related to purine metabolism were enriched. Therefore, we analyzed the correlation between these flora and purine metabolites, and found that gout related flora can significantly regulate purine metabolites. This exploratory study indicates that verbenalin exert protective effects against gout and UA metabolic dysfunction. These benefits may be associated with the modulation of specific flora, the preservation of intestinal barrier function, and the regulation of metabolic pathways.

We screened for biomarkers by multivariate statistical analysis of urinary metabolites collected from samples. Urine metabolomics analysis revealed that 462 differential metabolites were associated with gout amelioration, mainly Maltotetraose, Aloesin, Maltotriose, and Pseudo-Hydroxyalprazolam, as well as major metabolic pathways, such as nucleotide metabolism and purine metabolism. Most of the UA in the body is mainly the end product of exogenous and endogenous purine metabolism, and purine is an important product of nucleotide metabolism, and HUA caused by disturbances in purine nucleotide metabolism is the main cause of gout (51). Under physiological conditions, UA is derived from enzymatic degradation of purine nucleotides. In the process of purine nucleotide metabolism, guanine nucleotide (GMP) is converted to guanine by nucleotidase, and guanine is converted to xanthine by guanine deaminase. adenine nucleotide (AMP) is converted into HX nucleic acid under the action of nucleotidase, and XOD eventually oxidizes HX to xanthine and xanthine to UA (52, 53). We found that verbenin significantly modulates purine nucleotide metabolism disorders associated with gout, suggesting that verbenin potentially reduces serum UA levels may by modulating the purine nucleotide metabolic imbalances associated with gout.

## 5 Limitations

It should be noted that the mechanism described in the article regarding the action of verbenalin through intestinal microorganisms is still a theoretical conclusion. Although the metabolomics data and the results of the microbiota analysis support this possibility, the direct causal relationship between the two has not been confirmed in this experimental system. Therefore, future research needs to conduct experiments such as antibiotic treatment or fecal microbiota transplantation, combined with targeted metabolomics analysis of intestinal contents, to directly verify the necessity and mediating role of the intestinal microbiota in regulating the host’s UA metabolism through verbenalin.Furthermore, although this study identifies a significant modulatory effect on the microbiome and urinary metabolome, the broader pharmacodynamic landscape remains to be fully mapped. Specifically, the temporal dynamics and bidirectional interplay between hepatic/renal impairment and urate metabolic dysfunction warrant further clarification to determine whether these pathological changes are primary drivers or secondary consequences.

## 6 Conclusion

In conclusion, our exploratory findings suggest that verbenalin help modulate purine nucleotide metabolic disturbances, potentially through the regulation of intestinal microbiota and UA-related metabolic proteins. Furthermore, verbenalin was observed to attenuate inflammatory responses in the gout rat model, by reinforcing the intestinal barrier and inhibiting inflammatory signaling pathways. These results offer preliminary insights that warrant further investigation into the potential role of verbenalin in gout management.

## Data Availability

The raw data supporting the conclusions of this article will be made available by the authors, without undue reservation.
